# Biomineralized Nanocomposite‐Integrated Microneedle Patch for Combined Brachytherapy and Photothermal Therapy in Postoperative Melanoma Recurrence and Infectious Wound Healing

**DOI:** 10.1002/advs.202414468

**Published:** 2025-02-04

**Authors:** Peng Liu, Lu Hao, Jessica C. Hsu, Ming Zhou, Zhisheng Luo, Ying Peng, Weibo Cai, Shuo Hu

**Affiliations:** ^1^ Department of Nuclear Medicine Xiangya Hospital Central South University No. 87 Xiangya Road Changsha Hunan 410008 China; ^2^ Key Laboratory of Biological Nanotechnology NHC. No. 87 Xiangya Road Changsha Hunan 410008 China; ^3^ Departments of Radiology and Medical Physics University of Wisconsin‐Madison Madison WI 53705 USA; ^4^ Xiangya School of Pharmaceutical Sciences Central South University Changsha Hunan 410013 China

**Keywords:** brachytherapy, infectious wound healing, local hyperthermia, melanoma, microneedle patch, postoperative recurrence

## Abstract

In the surgical management of malignant melanoma, incomplete tumor resection and large‐area cutaneous defects are major contributors to high locoregional recurrence and uncontrolled wound infections, resulting in poor prognosis and prolonged recovery times for patients. Herein, a versatile nanocomposite microneedle patch (referred to as GM‐Ag_2_S/Ca^32^P) is designed to simultaneously eliminate residual tumor post‐surgery and promote the healing of infectious wounds. This microneedle patch effectively penetrates subcutaneous tissues, delivering therapeutic payloads to infiltrating tumor cells and bacteria. The Ag_2_S/Ca^32^P nanocomposites encapsulated within the microneedle patch decompose in the acidic microenvironment of tumors and bacterial biofilms, releasing radioactive ^32^P and Ag_2_S nanodots, which enhance tumor eradication and bacteria killing through synergistic brachytherapy and photothermal therapy (PTT). Moreover, the nanocomposite microneedle patch promotes scar‐free wound healing by reducing inflammation, and promoting granulation tissue formation, collagen deposition, and angiogenesis, thanks to localized hyperthermia, radiation, and the swelling and biodegradation of the microneedle matrices. This microneedle patch‐based postoperative adjuvant therapy offers a comprehensive strategy for addressing melanoma recurrence and infectious wound healing, with promising potential for clinical application in postsurgical management.

## Graphical abstract

This study develops a versatile nanocomposite microneedle patch (GM‐Ag_2_S/Ca^32^P) to target residual tumors and promote wound healing post‐surgery. By delivering radioactive ^32^P and Ag_2_S nanodots, the patch enables synergistic brachytherapy and photothermal therapy to enhance tumor and bacteria elimination. Additionally, GM‐Ag_2_S/Ca^32^P promotes scar‐free wound healing by reducing inflammation, and promoting granulation tissue formation, collagen deposition, and angiogenesis, offering a promising approach for postoperative melanoma management.

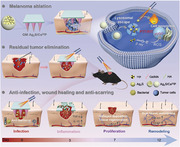

## 1 Introduction

Cutaneous melanoma, one of the most aggressive forms of skin cancer, affects over a million individuals worldwide each year, posing a significant threat to both health and quality of life.^[^ ^1^ ^]^ Clinically, the predominant treatment for melanoma is the surgical resection of superficial tumors and surrounding normal tissues, with ≈80% to 90% of early‐stage melanoma patients undergoing surgery.^[^ ^2^, ^3^ ^]^ However, incomplete surgical excision often leaves behind infiltrating but asymptomatic tumor cells, resulting in a significantly higher risk of local recurrence or distant metastasis.^[^ ^4^ ^]^ Additionally, patients with compromised immune systems, coupled with large‐area cutaneous defects after tumor surgery, increase the risk of bacterial infections and chronic inflammatory wounds that may pose a threat to their survival.^[^ ^5^, ^6^ ^]^ Therefore, a comprehensive treatment strategy that simultaneously eradicates remaining melanoma cells, provides robust infection control and accelerates wound healing is crucial to improving the long‐term survival rate and overall life quality after surgical removal of melanoma.

To eliminate residual melanoma cells after surgery to prevent local recurrence or metastasis, adjuvant radiotherapy is commonly applied in clinical practice.^[^ ^7^ ^]^ Nevertheless, traditional external beam radiotherapy (EBRT) can be ineffective against melanoma, which often shows a lack of sensitivity to radiation.^[^ ^8^, ^9^ ^]^ On the other hand, brachytherapy, a more localized form of radiotherapy, has gained extensive clinical use due to its ability to deliver high doses of radiation with precision, while minimizing damage to surrounding healthy tissues. This technique is especially effective for treating recurrent melanoma or in regions where other treatments prove insufficient or impractical.^[^ ^10^, ^11^ ^]^ Radioactive phosphorus‐32 (^32^P), a beta emitter with moderate tissue penetration and a half‐life of 14.3 days, is well‐suited for treating localized, superficial tumors.^[^ ^12^ ^]^ When applied as a radioactive skin patch, ^32^P can effectively target and eradicate residual tumor cells, serving as an adjuvant therapy.^[^ ^13^ ^]^ Notably, ^32^P could promote a smoother healing process by regulating fibroblast activity and preventing excessive collagen buildup, showing a wide range of clinical applications in wound healing and scar inhibition.^[^ ^14^ ^]^ While ^32^P‐based brachytherapy is effective in eradicating tumor cells, it does not address the risk of infection, which is a major factor contributing to inflammation and delayed wound healing. Photothermal therapy (PTT), which converts light energy into heat, has emerged as a promising approach for combating wound infection owing to its high selectivity, minimal side effects, and precise spatiotemporal control.^[^ ^15^ ^]^ Moreover, PTT‐induced hyperthermia can hinder cancer cell DNA repair processes and improve blood flow and oxygenation, thereby enhancing the therapeutic effectiveness of radiotherapy in melanoma treatment.^[^ ^16^ ^]^ In recent years, various photothermal agents, including organic agents, metal‐based nanomaterials, carbon‐based nanomaterials, and organic/inorganic nanocomposites, have been explored to destroy bacterial integrity and provide anti‐biofilm therapy.^[^ ^17^, ^18^ ^]^ Among them, Ag_2_S nanodots have excellent photothermal conversion efficiency and resistance to photobleaching, making them ideal for various biomedical applications.^[^ ^19^, ^20^, ^21^ ^]^

Localized delivery of photothermal agents to tumor tissue can enhance hyperthermia‐induced tumor damage while minimizing skin phototoxicity.^[^ ^6^, ^22^ ^]^ However, the physiological barrier of the skin often hampers these agents from reaching deeper tissue layers, posing significant challenges for the localized PTT of melanoma.^[^ ^23^ ^]^ Microneedle‐based patches have attracted widespread interest as a transdermal drug delivery method, valued for their ease of use, painless application, uniform drug distribution, and minimally invasive nature.^[^ ^24^, ^25^ ^]^ Additionally, the matrix of microneedle patches, which includes hyaluronic acid (HA) and gelatin, has characteristics similar to those of the extracellular matrix and can repair injured tissues and accelerate regeneration.^[^ ^26^ ^]^ Various microneedle platforms have been developed for cancer treatment, infection control, and wound healing.^[^ ^27^, ^28^, ^29^ ^]^ However, multifunctional microneedle patches capable of mitigating melanoma recurrence, wound infection, and scar formation remain underexplored.

**Scheme 1:**
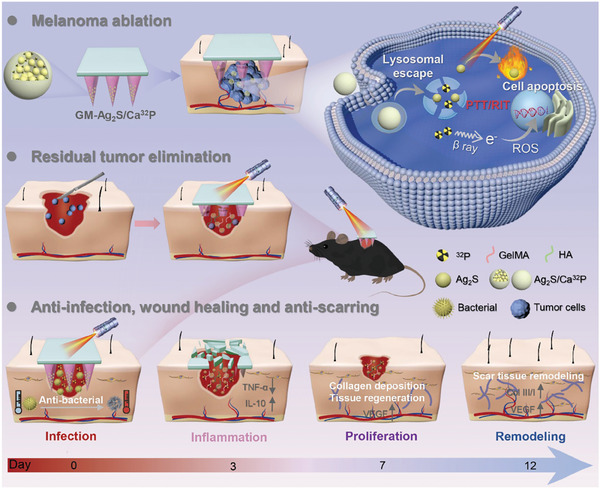
Schematic illustrating the application of the GM‐Ag_2_S/Ca^32^P microneedle patch for treating postoperative melanoma recurrence and facilitating infectious wound healing. The GM‐Ag_2_S/Ca^32^P microneedle patch effectively eliminates residual melanoma cells and bacteria through a synergistic combination of brachytherapy and PTT. In the subsequent healing process, it alleviated inflammation, facilitated granulation tissue formation, and enhanced collagen deposition and angiogenesis, thereby achieving scar‐free healing of infectious wounds.

In this study, we developed a versatile nanocomposite microneedle patch that is functional for combined brachytherapy and PTT, thus providing an enhanced adjuvant therapy for postoperative melanoma recurrence and infectious wound healing (**Scheme** **1**). Here Ag_2_S nanodots were incorporated into ^32^P‐labeled calcium phosphate (Ca^32^P) nanoparticles via a two‐step biomineralization process, obtaining Ag_2_S/Ca^32^P nanocomposites. Then, we engineered a microneedle patch, using methacrylate gelatin (GelMA) as the needle tips and HA as the backing layer, to deliver Ag_2_S/Ca^32^P to infiltrating tumor cells and bacterial biofilms. The as‐fabricated GM‐Ag_2_S/Ca^32^P microneedle patch effectively penetrated subcutaneous tissues and transported Ag_2_S/Ca^32^P to target sites. Upon reaching the acidic microenvironment of tumors and bacterial biofilms, the Ag_2_S/Ca^32^P nanocomposites decomposed, releasing radioactive ^32^P and Ag_2_S nanodots, thereby enhancing tumor cell elimination and bacterial killing through synergistic effects of brachytherapy and PTT. Moreover, the combination of localized hyperthermia, radiation, and the biodegradation of GelMA and HA facilitated scar‐free wound healing by reducing inflammation and promoting granulation tissue formation, collagen deposition, and angiogenesis. Therefore, the GM‐Ag_2_S/Ca^32^P microneedle patch thus represents a multi‐functional strategy for managing melanoma recurrence and infected wounds, demonstrating great prospects for post‐surgical clinical applications.

## 2 Results and Discussion

### 2.1 Fabrication of Ag_2_S/Ca^32^P Nanocomposites with pH‐Responsive and Photothermal Conversion Properties

**Figure 1:**
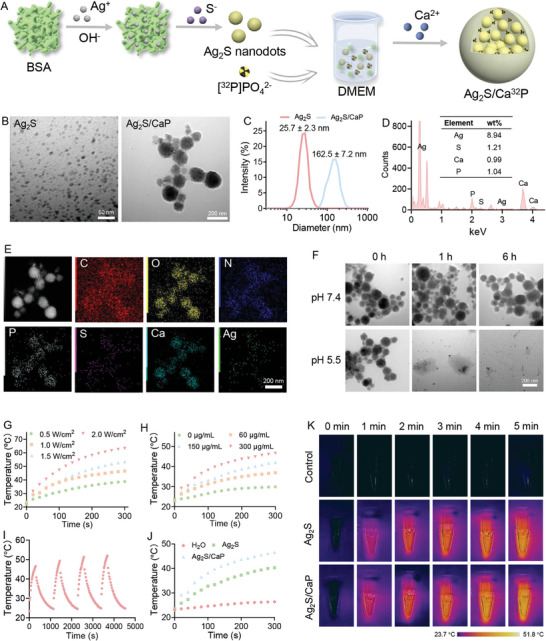
A) Schematic illustration of the preparation process for Ag_2_S/Ca^32^P nanocomposites. B) TEM images and C) hydrodynamic diameter measurements of Ag_2_S and Ag_2_S/CaP. D) EDS analysis and E) elemental mapping of Ag_2_S/CaP. F) TEM images of Ag_2_S/CaP after incubation in neutral (pH 7.4) and acidic (pH 5.5) conditions. G) Temperature‐increment profiles of Ag_2_S/CaP when irradiated using various laser power densities for 300 s. H) Concentration‐dependent temperature profiles of Ag_2_S/CaP when irradiated using the same laser power density. I) Photothermal conversion cycling test of Ag_2_S/CaP. J) Heating curves and K) real‐time infrared thermal images of Ag_2_S and Ag_2_S/CaP under irradiation.

The Ag_2_S/CaP nanocomposites were prepared using a two‐step biomineralization method (**Figure** **1**A). First, the BSA solution was mixed with AgNO_3_ for Ag^[+]^ absorption onto BSA via electrostatic interactions. The initial step of biomineralization was triggered by adjusting the pH to 12 and adding Na_2_S to allow nucleation, crystal growth, and the formation of Ag_2_S nanodots. Transmission electron microscopy (TEM) imaging showed that the Ag_2_S nanodots had a uniform morphology with particle sizes below 10 nm (Figure 1B). The hydrodynamic diameter measured by dynamic light scattering (DLS) was 25.7 nm, which can be attributed to the presence of the protein corona on these nanodots (Figure 1C). Afterward, Ag_2_S nanodots were added to BSA‐containing Dulbecco's Modified Eagle's Medium (DMEM). BSA acted as chelating agents, binding to Ca^2+^ ions and elevating the local supersaturation, which created nucleation sites for the latter step of biomineralization. This process allowed Ca^2+^ to react with anionic phosphate in the DMEM, leading to the formation of small crystallites that gradually grew and densified into stable Ag_2_S/CaP nanocomposites. The Ag_2_S/CaP was spherical with an average size of 162.5 nm, and a large amount of Ag_2_S nanodots could be seen inside the CaP nanoparticles (Figure 1B,C). Energy dispersive X‐ray spectroscopy (EDS) and elemental mapping showed the copresence and distribution of Ag, S, Ca, and P elements in the nanocomposites, further confirming the successful fabrication of Ag_2_S/CaP (Figure 1D,E). The fabrication of Ag_2_S/Ca^32^P for radionuclide ^32^P labeling followed a procedure similar to that of Ag_2_S/CaP, with the addition of a trace amount of H[^32^P]PO_4_^2−^, as previously reported.^[^ ^30^ ^]^ To test the degradation of Ag_2_S/CaP in the acidic tumor and bacterial biofilm microenvironments, the nanocomposites were incubated under different pH conditions and analyzed via TEM. Ag_2_S/CaP displayed minimal morphological change over 6 h in a pH 7.4 buffer solution, indicating its excellent stability under physiological conditions. Notably, when incubated in an acidic condition (pH 5.5), the outer layer of Ag_2_S/CaP rapidly degraded, resulting in the release of Ag_2_S nanodots (Figure 1F).

Previous studies have convinced us that Ag_2_S nanodots exhibit a strong photothermal effect due to their favorable near‐infrared (NIR) absorbance.^[^ ^19^ ^]^ The photothermal conversion capacity of Ag_2_S/CaP was measured under 808 nm NIR laser irradiation. Temperature elevations were found to be positively correlated with both the irradiation power density from 0.5 to 2 W cm^−2^ and the concentration of Ag_2_S/CaP from 0 to 300 µg mL^−1^ (Figure 1G,H). Specifically, Ag_2_S/CaP at a concentration of 300 µg mL^−1^ induced a temperature increase of ≈23 °C in 300 s under 1 W cm^−2^ laser irradiation. Furthermore, Ag_2_S/CaP showed excellent photostability, maintaining its photothermal performance across four cycles of laser on/off (Figure 1I). When compared to Ag_2_S nanodots, Ag_2_S/CaP displayed superior photothermal conversion activities, as shown by heating curves and real‐time infrared thermal images (Figure 1J,K). These results confirmed that Ag_2_S/CaP possesses outstanding photothermal performance, making it an ideal candidate for tumor ablation and bacterial eradication post‐surgery.

### 2.2 Cellular Uptake of Ag_2_S/Ca^32^P and In Vitro Cytotoxicity via Synergistic Brachytherapy/PTT

**Figure 2:**
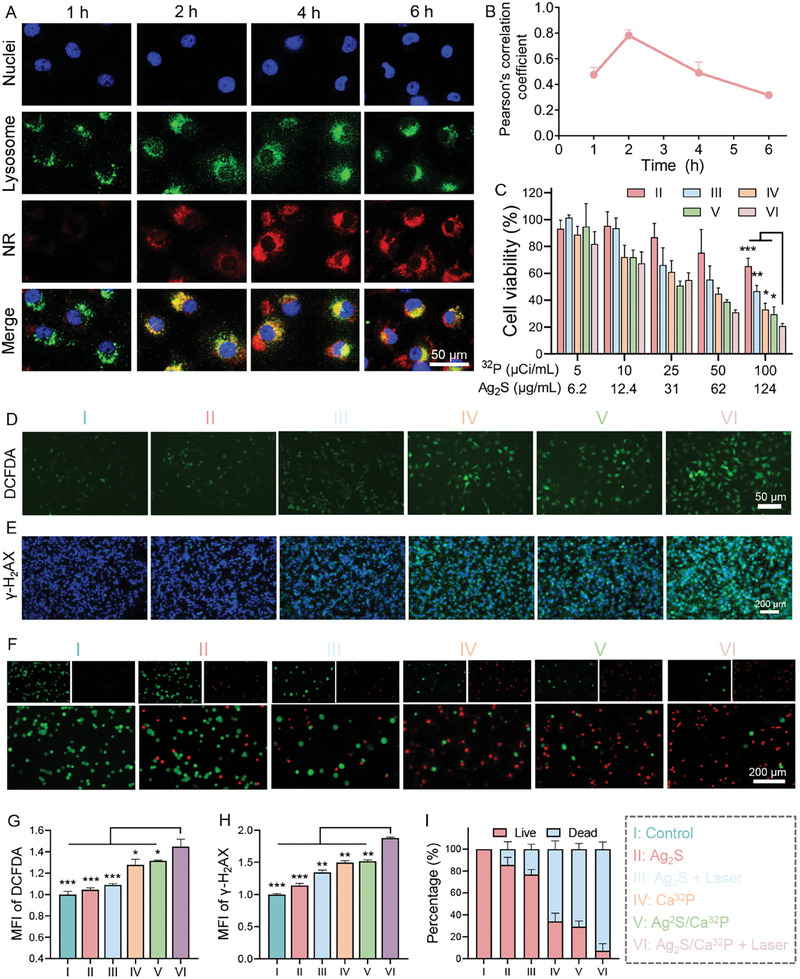
A) Intracellular localization of the NR‐labeled Ag_2_S/CaP and lysosomal escape in B16F10 cells. B) Time‐dependent evolution of Pearson's correlation coefficients between NR and LysoTracker signals (*n* = 3). C) Viability of B16F10 cells treated with various formulations, with and without laser irradiation (*n* = 5). D) Fluorescence images and G) quantitative analysis of B16F10 cells stained with DCFH‐DA after various treatments (*n* = 3). E) γ‐H_2_AX fluorescence images (blue: DAPI, green: γ‐H_2_AX) and H) quantitative analysis of B16F10 cells after different treatments (*n* = 3). F) Fluorescence images and I) quantitative analysis of live/dead cells after various treatments (*n* = 3). ^*^ *p* < 0.05, ^**^ *p* < 0.01, ^***^ *p* < 0.001.

After investigating the physicochemical properties and functions of Ag_2_S/Ca^32^P, we conducted cellular studies to further assess its therapeutic efficacy against tumor cells. Initially, the cellular uptake behavior of the nanocomposites was explored using confocal laser scanning microscopy (CLSM). As presented in **Figure** **2**A, B16F10 melanoma cells were incubated with Nile red (NR)‐labeled Ag_2_S/CaP for 1, 2, 4, and 6 h. Red fluorescence signal within the cells increased over time, reaching a maximum at 4 h, demonstrating effective cellular uptake of nanocomposites. To study lysosomal escape, lysosomes were stained with Lyso‐Tracker Green (green), and Pearson's correlation coefficients between the nanocomposites and lysosomes were quantified to determine the escape rate. It was found that the nanocomposites were internalized by the cells and initially trapped in lysosomes during the first 2 h, as evidenced by an increase in the coefficient (Figure 2B). However, when the incubation time was extended from 2 to 6 h, the coefficient reduced significantly from 0.75 to 0.31, suggesting that the nanocomposites gradually escaped from the lysosomes. The underlying mechanisms facilitating lysosomal escape were likely proton consumption and nanoparticle disintegration.^[^ ^31^ ^]^

After internalization and entry into the cytoplasm, Ag_2_S/Ca^32^P emits β‐ray, generating ionized atoms that can damage DNA directly or produce ROS in the presence of O_2_. We then detected the intracellular ROS levels using a fluorogenic probe (DCFH‐DA). Strong green fluorescence was observed in cells treated with Ca^32^P or Ag_2_S/Ca^32^P (Figure 2D,G), indicating substantial ROS production. Interestingly, Ag_2_S/Ca^32^P combined with laser irradiation showed even brighter fluorescence intensity than Ag_2_S/Ca^32^P alone, while Ag_2_S with laser irradiation had a negligible effect on intracellular ROS levels. This phenomenon suggested that photothermal heating amplifies ^32^P‐mediated ROS generation. Next, we evaluated DNA double‐strand damage in tumor cells using γ‐H_2_AX immunofluorescence staining. As expected, Ag_2_S/Ca^32^P combined with laser irradiation exhibited a stronger fluorescence signal compared to other groups, indicating more severe DNA breaks (Figure 2E,H). The combination of hyperthermia and ^32^P‐mediated radiation from Ag_2_S/Ca^32^P is thus crucial for effective cancer cell elimination. To further assess cytotoxicity, we performed an MTT assay on B16F10 cells treated with the nanocomposites. Ag_2_S nanodots alone induced minimal toxicity (Figure 2C). However, cell viability in the Ag_2_S group was markedly reduced after laser irradiation, confirming the potent PTT effect induced by the Ag_2_S nanodots. Both Ca^32^P and Ag_2_S/Ca^32^P showed concentration‐dependent cytotoxicity, benefiting from ^32^P‐mediated radiation. Notably, Ag_2_S/Ca^32^P combined with laser irradiation exhibited significantly enhanced cytotoxicity, causing over 80% cell death at higher concentrations. Fluorescence imaging of cells co‐stained with Calcein AM (live/green) and propidium iodide (dead/red) further confirmed substantial cancer cell death in the Ag_2_S/Ca^32^P + Laser group (Figure 2F,I). These results collectively highlight the excellent efficacy of the synergistic brachytherapy and PTT approach in cancer treatment.

### 2.3 Fabrication of GM‐Ag_2_S/Ca^32^P Microneedle Patch and Evaluation of Antibacterial Activity In Vitro

**Figure 3:**
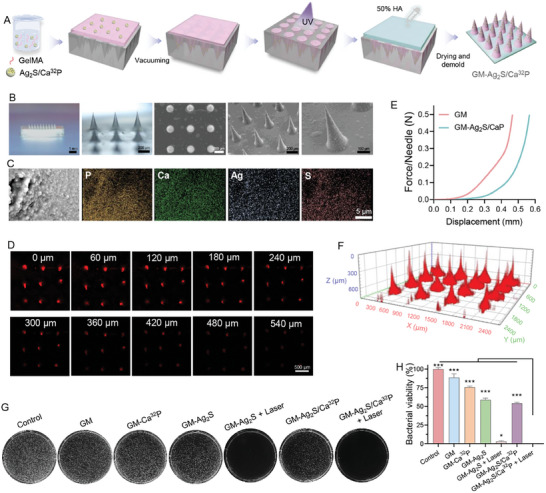
A) Schematic illustration of the fabrication process for the GM‐Ag_2_S/Ca^32^P microneedle patch. B) Photograph, optical microscopy, and SEM images of GM‐Ag_2_S/CaP. C) Elemental mapping of the needle surface of GM‐Ag_2_S/CaP. D) Fluorescence images and F) 3D reconstructed images of RhB‐labeled GM‐Ag_2_S/CaP. E) Force‐displacement curves of the microneedle patch under different applied forces. G) Agar plate images and H) bacterial viability of *S. aureus* after various treatments (*n* = 3). ^*^ *p* < 0.05, ^***^ *p* < 0.001.

Furthermore, the GM‐Ag_2_S/Ca^32^P microneedle patch was fabricated using a micro‐molding and ultraviolet (UV)‐induced polymerization technique. Briefly, the Ag_2_S/Ca^32^P was incorporated into methacrylate gelatin (GelMA) to obtain a homogeneously dispersed pre‐gel solution. This solution was then placed into microneedle molds, where it solidified into needle tips upon exposure to UV light. Next, a hyaluronic acid (HA) solution was added to the base, and after drying and demolding, a microneedle patch with Ag_2_S/Ca^32^P‐loaded GelMA tips and HA backing layer arranged in a 10 × 10 array was produced (**Figure** **3**A). Optical microscopy and scanning electron microscopy (SEM) confirmed the well‐defined 3D structure of GM‐Ag_2_S/CaP, characterized by sharp tips and uniform size, with a height of 550 µm (Figure 3B). SEM‐EDS mapping analysis revealed a uniform distribution of P, Ca, Ag, and S elements within the needle tips, demonstrating the successful incorporation of Ag_2_S/CaP into the microneedles (Figure 3C). To further visualize the microneedle patch, rhodamine B (RhB)‐loaded Ag_2_S/CaP were integrated into the needle tips and observed using CLSM. Fluorescence imaging at various depths, along with 3D reconstructions demonstrated even distribution of payloads throughout the tips (Figure 3D,F). Moreover, force‐displacement curves were obtained by applying pressure to the microneedles, and the smooth, continuous curves for GM and GM‐Ag_2_S/CaP showed no needle damage under pressures up to 0.5 N/needle (Figure 3E). At a displacement of 400 µm, the fracture forces for GM and GM‐Ag_2_S/CaP were 0.24 and 0.08 N per needle, respectively, both exceeding the reported minimum effective force (0.045 N per needle) required for skin penetration,^[^ ^32^ ^]^ thus confirming the excellent mechanical strength of GM‐Ag_2_S/CaP for piercing the skin and scalable for in vivo applications.

In clinical settings, bacterial infection of a wound following a surgical cancer treatment is a common complication that can delay healing, promote tumor recurrence, and increase mortality rates. Therefore, the development of safe and efficient formulations to prevent postoperative bacterial infection is of great concern. To address this challenge, we subsequently assessed the antibacterial efficacy of GM‐Ag_2_S/Ca^32^P against *S. aureus*. The GM‐Ag_2_S and GM‐Ag_2_S/Ca^32^P microneedle patches demonstrated potent antibacterial activity against *S. aureus* in the presence of laser irradiation, achieving killing efficiencies close to 100% (Figure 3G,H). In contrast, groups without laser irradiation showed relatively weak antibacterial activity (≈45%). Additionally, both the GM and GM‐Ca^32^P groups demonstrated minimal antibacterial effects, with inhibition efficiencies below 30%. These findings suggest that the antibacterial properties of GM‐Ag_2_S/Ca^32^P are driven by Ag_2_S‐induced PTT, which plays a crucial role in facilitating the healing of infected wounds.

### 2.4 Effective Skin Penetration of GM‐Ag_2_S/Ca^32^P for Ablation of B16F10 Melanoma Tumors

**Figure 4:**
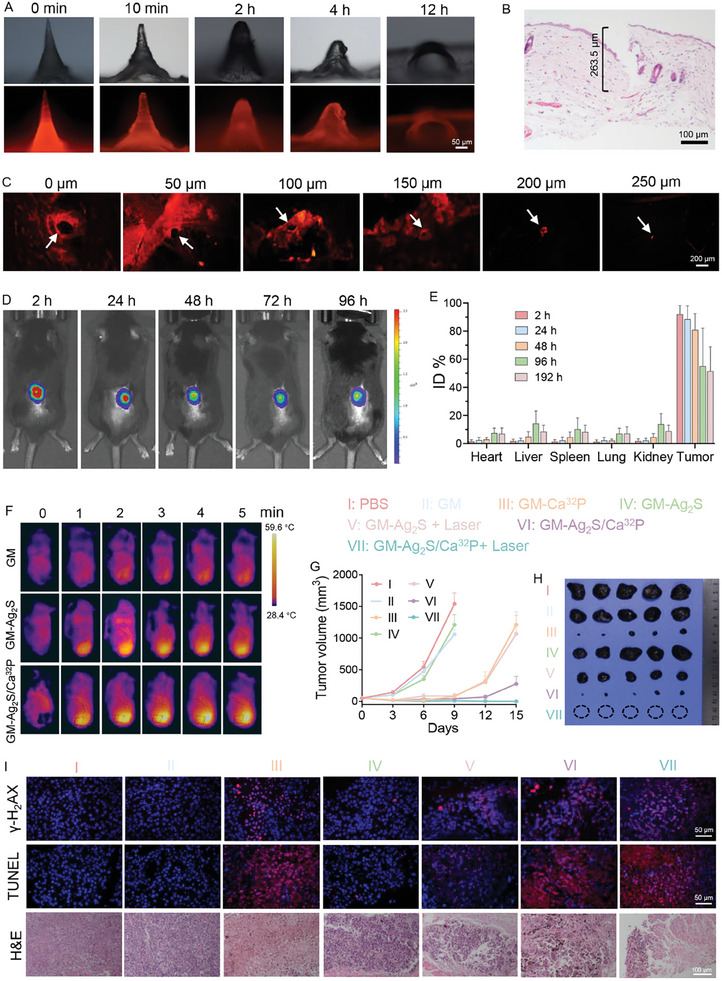
A) Optical and fluorescence imaging of RhB‐loaded GM‐Ag_2_S/Ca^32^P after microneedle insertion into mouse skin. B) H&E staining of mouse skin after administration of microneedle patch. C) Fluorescence imaging of mouse skin at different depths after application of RhB‐loaded GM‐Ag_2_S/Ca^32^P. D) In vivo fluorescent imaging and E) radioactive ^32^P biodistribution at various time points post‐insertion of ICG‐loaded GM‐Ag_2_S/Ca^32^P (*n* = 4). F) Real‐time infrared thermal images of mice undergoing different treatments. G) Tumor volume of mice after various treatments (*n* = 5). H) Photographs illustrating the gross tumor volume post‐treatment across various groups. I) Tumor section analysis via immunofluorescence staining (γ‐H_2_AX, TUNEL) and H&E staining.

Considering the excellent mechanical strength of the microneedle patch, we then explored its penetration and biodistribution after insertion into mouse skin. RhB‐loaded GM‐Ag_2_S/Ca^32^P was applied to the skin of mice for various durations, and the remaining needles were observed using optical and fluorescence microscopy. The needle tips were found to dissolve within 2 h of insertion, with complete dissolution occurring by 12 h (**Figure** **4**A). H&E staining revealed that GM‐Ag_2_S/Ca^32^P generated microchannels in the skin (Figure 4B), and the skin returned to normal 20 min after the patch was removed (Figure S1, Supporting Information). Furthermore, fluorescence microscopy revealed that RhB diffused to a depth exceeding 200 µm within the skin (Figure 4C). These findings underscore the excellent transdermal capability of the GM‐Ag_2_S/Ca^32^P microneedle patch and its effectiveness in delivering payloads to the local skin site.

Owing to the inherent targeting capability of microneedles toward superficial tumors, we evaluated the topical accumulation and biodistribution of GM‐Ag_2_S/Ca^32^P in a subcutaneous B16F10 melanoma mouse model. The ICG‐loaded GM‐Ag_2_S/Ca^32^P was inserted into the tumor sites and removed after 2 h. In vivo, fluorescence imaging revealed that ICG‐loaded GM‐Ag_2_S/Ca^32^P persisted in the skin for over 96 h (Figure 4D). Additionally, the radioactivity of ^32^P in major organs and tumor tissue was quantified using a gamma counter. A concentrated signal of radionuclide was detected in the tumors after the local application of GM‐Ag_2_S/Ca^32^P (Figure 4E). On the other hand, major organs, including the heart, liver, spleen, lung, and kidneys, showed negligible radioactivity throughout the 96‐h monitoring period, suggesting the prolonged tumor accumulation of GM‐Ag_2_S/Ca^32^P. This sustained localization is promising for enhanced anticancer efficacy with reduced systemic toxicity.

Then, the in vivo antitumor effects of GM‐Ag_2_S/Ca^32^P were investigated in B16F10 tumor‐bearing mice. The microneedle patch was directly applied to the tumor site, followed by 5 min of laser irradiation. Compared to the control GM microneedle patch, the temperature in the GM‐Ag_2_S and GM‐Ag_2_S/Ca^32^P groups rapidly increased to 50 °C and remained stable during the 5‐min irradiation period, surpassing the tumor damage threshold (Figure 4F). Based on this, we randomly divided the tumor‐bearing mice into seven groups: PBS, GM, GM‐Ca^32^P, GM‐Ag_2_S, GM‐Ag_2_S + Laser, GM‐Ag_2_S/Ca^32^P, GM‐Ag_2_S/Ca^32^P + Laser. Compared to the control group, there was no obvious difference in tumor volume between the GM and GM‐Ag_2_S groups, with both groups exhibiting rampant tumor growth (Figure 4G,H). In contrast, tumor growth was slower in the GM‐Ca^32^P and GM‐Ag_2_S/Ca^32^P groups, indicating the tumoricidal potential of ^32^P due to its radiation effects. Similarly, the GM‐Ag_2_S + Laser group showed a significant reduction in tumor volume, attributed to the antitumor effects of PTT. However, these groups did not completely inhibit tumor growth, only delaying it temporarily. Significantly, the GM‐Ag_2_S/Ca^32^P + Laser group demonstrated marked tumor eradication. Immunofluorescence staining (γ‐H_2_AX, TUNEL) and H&E staining of tumor tissue further revealed extensive DNA damage, enhanced tumor apoptosis, and necrosis in the GM‐Ag_2_S/Ca^32^P + Laser group (Figure 4I; Figure S2, Supporting Information), highlighting the excellent synergistic antitumor effects of brachytherapy and PTT.

### 2.5 Inhibition of Tumor Recurrence by GM‐Ag_2_S/Ca^32^P in a Postoperative Melanoma Model

**Figure 5:**
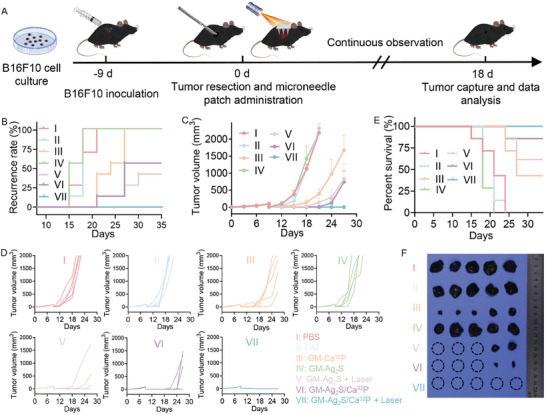
A) Schematic illustration of the treatment and monitoring schedule in a mouse model of incomplete tumor resection. B) Tumor recurrence rate, C) average tumor growth kinetics, and D) individual tumor growth profiles across different groups (*n* = 5). E) Survival period of mice in each group. F) Images of excised tumors on day 35.

Surgical procedures often fail to completely eliminate tumors, and residual tumor tissue frequently leads to recurrence, posing a difficulty in clinical cancer treatment. Given the exceptional antitumor efficacy of GM‐Ag_2_S/Ca^32^P, its ability to prevent postoperative tumor recurrence was evaluated. For this purpose, mice were treated with different microneedle patches following tumor resection (**Figure** **5**A). To ensure similar surgical outcomes, the wound size and amount of remaining tumor tissue were kept as uniform and consistent as possible. All mice in the PBS, GM, and GM‐Ag_2_S groups suffered tumor recurrence within 20 days (Figure 5B). By comparison, the GM‐Ca^32^P, GM‐Ag_2_S/Ca^32^P, and GM‐Ag_2_S + Laser groups exhibited varying degrees of recurrence over the 35‐day monitoring period, indicating modest anti‐recurrence effects. Notably, none of the five mice treated with GM‐Ag_2_S/Ca^32^P + Laser showed any signs of postoperative tumor recurrence. Furthermore, the tumor growth curves indicated minimal tumor growth in the GM‐Ag_2_S/Ca^32^P + Laser group (Figure 5C,D). Photographs of excised tumor tissues further confirmed the effectiveness of this treatment in complete tumor elimination (Figure 5F). Mice treated with GM‐Ag_2_S/Ca^32^P + Laser had a markedly prolonged survival period, surviving for more than 35 days (Figure 5E).

The in vivo biocompatibility of GM‐Ag_2_S/Ca^32^P microneedle patch was further evaluated. A slight reduction in body weight was observed in all groups on the third‐day post‐surgery. However, throughout the treatment, the body weight of mice gradually increased, with no significant changes overall (Figure S3, Supporting Information). H&E staining of major organs, including the heart, liver, spleen, lung, and kidneys, showed no notable pathological changes in any group (Figure S4, Supporting Information). Additionally, blood biochemistry parameters (ALT, AST, BUN, CREA) remained close to the normal range and showed no significant differences between groups (Figure S5, Supporting Information). These results confirmed that the GM‐Ag_2_S/Ca^32^P microneedle patch is a biocompatible platform for preventing postoperative tumor recurrence.

### 2.6 In Vivo Evaluation of GM‐Ag_2_S/Ca^32^P for Infection Control and Wound Closure

**Figure 6:**
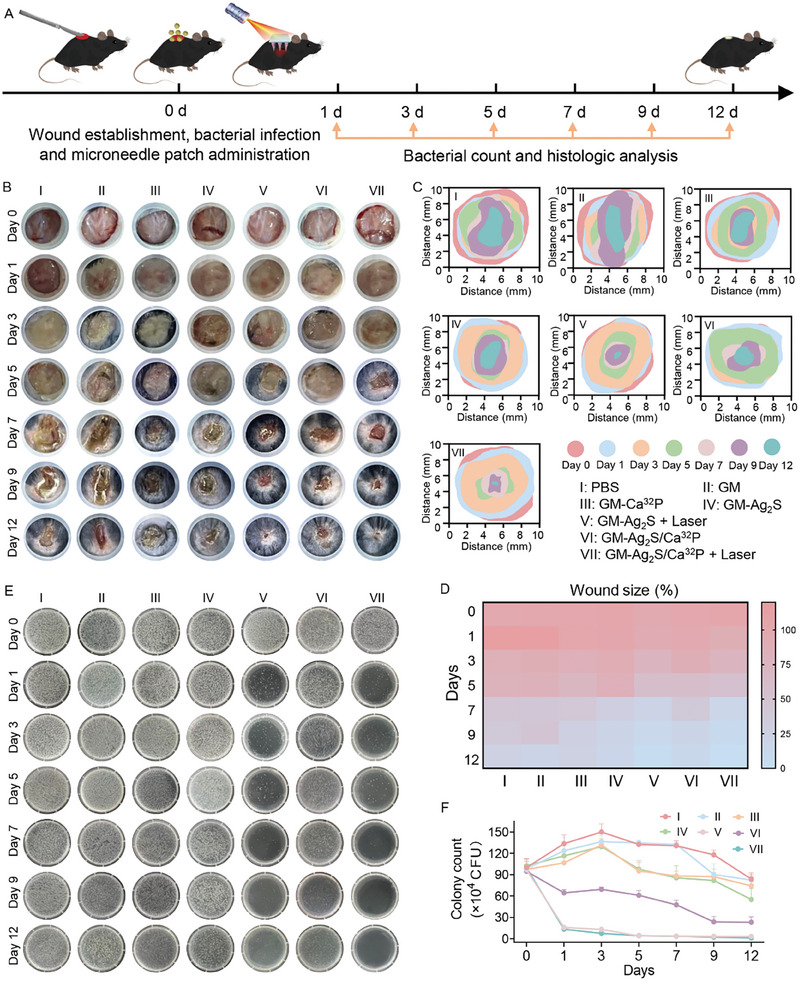
A) Schematic illustration of the construction and treatment process of a full‐thickness *S. aureus*‐infected wound model. B) Representative photographs of mouse skin wounds, C) changes in wound size through simulation, and D) quantified heatmap analysis of infected wounds throughout the treatment period (*n* = 3). E) Photographs of agar plates showing *S. aureus* colonies collected from infected tissues during treatment, and F) corresponding quantitative analysis of colony growth (*n* = 3).

Full‐thickness skin defects and large open wounds following surgical tumor resection greatly elevate the risk of infection, complicating the wound‐healing process. Therefore, the antibacterial efficacy and skin tissue regeneration ability of GM‐Ag_2_S/Ca^32^P microneedle patch is important for promoting wounded skin tissue recovery after surgery. We then assessed the infection‐impaired skin wound healing capacity of GM‐Ag_2_S/Ca^32^P using a full‐thickness *S. aureus*‐infected wound model. *S. aureus* was inoculated onto the wound surfaces, followed by the application of various microneedle patches, which were then irradiated with an 808 nm laser (**Figure** **6**A). The wound areas were photographed, and bacteria from the wound sites were collected for culture. After three days of treatment, wounds in the PBS, GM, GM‐Ca^32^P, GM‐Ag_2_S, and GM‐Ag_2_S/Ca^32^P groups exhibited severe infection and inflammation, characterized by the presence of visible pus (Figure 6B). In contrast, the GM‐Ag_2_S + Laser and GM‐Ag_2_S/Ca^32^P + Laser groups showed reduced pus formation, and the number of *S. aureus* colonies isolated from these wounds was significantly lower than in the other groups (Figure 6E,F). By day 7, simulated wound size changes and quantified heatmap analysis revealed rapid healing in the GM‐Ag_2_S + Laser and GM‐Ag_2_S/Ca^32^P + Laser groups, with the *S. aureus* viability rate dropping below 5%, despite the presence of eschar (Figure 6C,D). By day 12, the GM‐Ag_2_S/Ca^32^P + Laser‐treated wound had healed most effectively, with a wound closure rate of 97.8%. Notably, this group also demonstrated the highest antibacterial activity against *S. aureus*, achieving a killing efficiency of up to 99%. Additionally, while the weight of mice in each group decreased during the first two days, likely due to the initial construction of the infection model, no significant abnormalities were observed in the following days (Figure S6, Supporting Information). These results demonstrate that the GM‐Ag_2_S/Ca^32^P microneedle patch, when combined with PTT, can significantly inhibit *S. aureus* infection, thereby promoting wound healing.

### 2.7 Wound Healing and Anti‐Scarring Effects of GM‐Ag_2_S/Ca^32^P via Histological Evaluation

**Figure 7:**
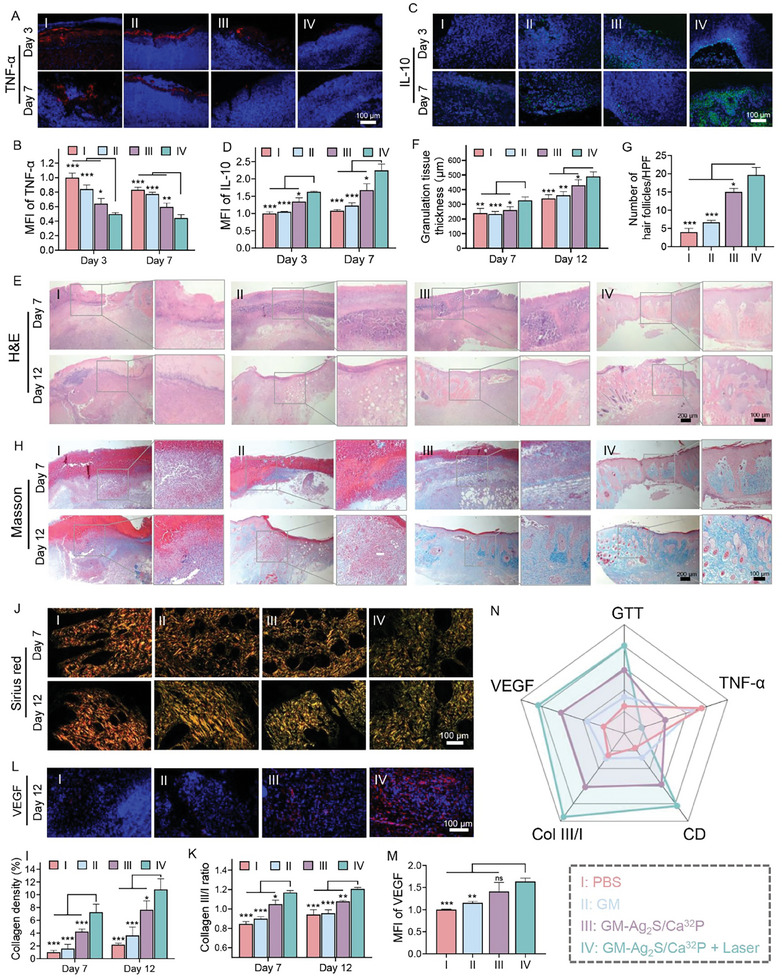
Immunofluorescence staining of A) TNF‐α and B) IL‐10 in skin wound tissue collected on days 3 and 7 from the different treatment groups (*n* = 3). Quantitative analysis of C) TNF‐α and D) IL‐10 expression levels following various treatments (*n* = 3). E) H&E staining and F) quantitative analysis of GTT in various groups on days 7 and 12 (*n* = 3). G) Number of hair follicles observed in highly magnified H&E‐stained tissue sections (n = 3). H) Masson's Trichrome staining and I) collagen density of wounded skin tissue on days 7 and 12 from various treated groups (*n* = 3). J) Sirius red staining and K) collagen III/I ratio of tissue sections on days 7 and 12 (*n* = 3). Type I collagen appears in red/orange, and type III collagen in yellow/green. L) Immunofluorescence staining and M) quantitative analysis of VEGF in wound tissue collected on day 12 (*n* = 3). N) Radar chart comparing wound healing and anti‐scarring effects of the various treatments. ^*^ *p* < 0.05, ^**^ *p* < 0.01, ^***^ *p* < 0.001.

Histological analysis was further conducted to evaluate the wound healing and anti‐scarring effects of GM‐Ag_2_S/Ca^32^P. Theoretically, wound healing is a complex and sequential process comprising four main phases: infection, inflammation, proliferation, and remodeling (Scheme 1).^[^ ^33^, ^34^ ^]^ Initially, infection occurs when pathogens invade the wound site, prompting an immune response aimed at eliminating them. During this phase, bacteria can proliferate in the wound, causing the secretion of inflammatory factors such as cytokines and chemokines to combat infection.^[^ ^35^ ^]^ However, an overactive inflammatory response delays the transition to the proliferation phase, impairing tissue regeneration and potentially leading to chronic wounds or excessive scarring.^[^ ^36^ ^]^ To investigate the extent of inflammation in wounded skin tissue, immunofluorescence analysis was conducted to evaluate the levels of tumor necrosis factor‐α (TNF‐α) and interleukin (IL‐10). Intense red fluorescence of TNF‐α was observed in PBS and GM groups on days 3 and 7, indicating severe inflammation (**Figure** **7**A,B). The expression of TNF‐α was obviously reduced in both GM‐Ag_2_S/Ca^32^P and GM‐Ag_2_S/Ca^32^P + Laser groups, with the lowest levels observed in GM‐Ag_2_S/Ca^32^P + Laser group. Conversely, IL‐10, a typical anti‐inflammatory factor, was drastically upregulated in the GM‐Ag_2_S/Ca^32^P + Laser group (Figure 7C,D), demonstrating its excellent antibacterial capability for eradicating infection and restraining inflammation in wounded skin tissue.

The subsequent proliferation phase involves fibroplasia, epithelialization, and wound contraction. In this phase, the granulation tissue forms as fibroblasts deposit collagen and extracellular matrix, providing a scaffold for new tissue formation. Concurrently, epithelial cells migrate to cover the wound, and myofibroblasts contract the wound edges to reduce its size.^[^ ^37^, ^38^ ^]^ We then performed H&E staining to assess the granulation tissue formation. As expected, the granulation tissue thickness (GTT) of GM‐Ag_2_S/Ca^32^P + Laser group was significantly greater than in the other groups on both days 7 and 12, exhibiting the highest skin integrity among the groups (Figure 7E,F). Notably, hair follicles and cutaneous appendages were evident in the GM‐Ag_2_S/Ca^32^P + Laser group on day 12 (Figure 7E,G). Masson Trichrome staining further revealed that the GM‐Ag_2_S/Ca^32^P + Laser group had higher collagen density (CD) compared to the other groups on days 7 and 12 (Figure 7H,I).

The final step in wound healing is tissue remodeling, during which collagen fibers and the extracellular matrix are reorganized to improve tissue strength and functionality, resulting in scar formation. However, an excessive inflammatory response can lead to severe hypertrophic scars, posing challenges in wound management and affecting the physical and mental health of patients.^[^ ^39^ ^]^ During wound healing, the balance between type I and type III collagen is crucial for regulating fiber formation and is responsible for scar tissue remodeling. Type I collagen contributes to the strength and structural integrity of healing tissues, while type III collagen provides initial support and flexibility.^[^ ^40^ ^]^ To assess collagen composition in the skin wound, we performed Sirius red staining. The ratio of collagen III to collagen I (Col III/I) in both GM‐Ag_2_S/Ca^32^P and GM‐Ag_2_S/Ca^32^P + Laser groups significantly increased on days 7 and 12 (Figure 7J,K), indicating ongoing tissue remodeling, with the wound becoming more organized and resembling the surrounding healthy tissues. The anti‐scarring effects can be attributed to ^32^P‐based brachytherapy, which is a well‐established clinical method for effectively suppressing the formation of hypertrophic scars.^[^ ^14^ ^]^ Moreover, angiogenesis, the formation of new blood vessels, was apparent during both the proliferation and remodeling phases, ensuring adequate blood supply to the healing tissue.^[^ ^41^ ^]^ The GM‐Ag_2_S/Ca^32^P + Laser group showed higher vascular endothelial growth factor (VEGF) levels on day 12 compared to other groups, indicating enhanced revascularization (Figure 7L,M).

We also compiled a summarized profile of the effects of TNF‐α levels, GTT, CD, Col III/I, and VEGF levels on wound healing and anti‐scarring outcomes (Figure 7N). The results indicated that GM‐Ag_2_S/Ca^32^P, when exposed to laser irradiation, facilitates scar‐free wound healing by inhibiting inflammation and promoting granulation tissue formation, collagen deposition, and angiogenesis. Specifically, the Ag_2_S nanodots released from GM‐Ag_2_S/Ca^32^P effectively combat infection through photothermal ablation under laser irradiation, facilitating the transition from the chronic inflammation phase to the proliferation phase. Additionally, the GelMA and HA matrix with its tissue regeneration properties, serves as a biological barrier, accelerating wound healing.^[^ ^42^ ^]^ The radioactive ^32^P not only plays a role in antibacterial and anti‐inflammatory, but also prevents excessive scarring. Therefore, the GM‐Ag_2_S/Ca^32^P microneedle patch demonstrates significant potential for managing bacterial infections in skin wounds and minimizing scar formation.

## 3 Conclusion

In summary, we have successfully developed a biocompatible nanocomposite microneedle patch (GM‐Ag_2_S/Ca^32^P) for postoperative melanoma recurrence prevention, infection inhibition, and scar‐free wound healing. Upon penetration and dissolution into subcutaneous tissues, the Ag_2_S/Ca^32^P nanocomposites degraded when exposed to the acidic tumor and bacterial biofilm microenvironment, releasing radioactive ^32^P and Ag_2_S nanodots. The pH‐responsive, local radiation and photothermal properties of Ag_2_S/Ca^32^P, coupled with the effective skin penetration of the microneedle patch, ensured efficient eradication of residual melanoma while minimizing systemic toxicity. Furthermore, the GM‐Ag_2_S/Ca^32^P microneedle patch deterred the occurrence of infection and inflammation, facilitated granulation tissue formation, enhanced collagen deposition, and stimulated angiogenesis, resulting in scar‐free wound healing. These findings highlight the promising application prospects of this microneedle patch for adjuvant melanoma treatment and the healing of infected wounds.

## 4 Experimental Section

### Materials

Bovine serum albumin (BSA), AgNO_3_, CaCl_2_, and Na_2_S·9H_2_O were acquired from Macklin Co., Ltd (Shanghai, China). Dialysis Membranes (MWCO:3500) were supplied by Yuanye Co., Ltd (Shanghai, China). Na_2_H[^32^P]PO_4_ was sourced from HTA Co., Ltd (Beijing, China). Nile red (NR), Rhodamine B (RhB), and Indocyanine Green (ICG) were procured from Aladin Co., Ltd (Shanghai, China). Lithium phenyl‐2,4,6‐trimethylbenzoylphosphinate (LAP) and gelatin methacryloyl (GelMA) were purchased from Engineering for Life Co., Ltd (Suzhou, China). Polydimethylsiloxane (PDMS) was obtained from Chipscreen Co., Ltd (Taizhou, China). Hyaluronic acid (HA, Mw≈10 kDa) was provided by MeilunBio Co., Ltd (Dalian, China). Hoechst 33 342, methyl thiazolyl tetrazolium (MTT), 4′, 6‐diamidino‐2′‐phenylindole (DAPI), 2′7'‐Dichlorofluorescin (DCFH‐DA) and Calcein AM/PI staining kit were obtained Beyotime Co., Ltd (Shanghai, China). The TUNEL staining kit was purchased from Elabscience Co., Ltd (Wuhan, China). Antibodies against TNF‐α and IL‐10 were obtained from Forevertek Biotechnology Co., Ltd (Changsha, China), while antibodies against γ‐H_2_AX and VEGF were purchased from Proteintech Co., Ltd (Wuhan, China).

### Preparation and Characterization of Ag_2_S/Ca^32^P Nanoparticles

BSA (500 mg) was dissolved into 14 mL of ultrapure water. Then, 2 mL AgNO_3_ (100 mM) solution was introduced, followed by the addition of NaOH to adjust the pH to 12. Subsequently, 4 mL Na_2_S·9H_2_O (100 mM) was added to the previously mentioned solution and incubated at 55 °C for 4 h. The solution was then dialyzed overnight to obtain Ag_2_S nanodots. Next, 200 mg BSA was dissolved into 18 mL DMEM and mixed with 2 mL of the prepared Ag_2_S nanodots, followed by adding 2 mCi Na_2_H[^32^P]PO_4_ (1 mCi mL^−1^) and 200 µL CaCl_2_ (1 M). After reacting overnight at 37 °C, Ag_2_S/Ca^32^P was isolated by centrifugation at 15 000 rpm for 20 min. The NR, RhB or ICG‐labeled Ag_2_S/Ca^32^P were synthesized by incorporating NR, RhB or ICG during the biomineralization process of Ca^32^P nanoparticles. Furthermore, the morphological features and elemental composition of nanoparticles were assessed employing transmission electron microscopy (TEM, Tecnai G2 F20, FEI, US). A Malvern Zetasizer (Nano ZS90, UK) was employed to ascertain the hydrodynamic diameters. The elements distribution was shown by Energy dispersive X‐ray spectroscopy (EDS, GeminiSEM 300, ZEISS, Germany).

### Photothermal Properties of Ag_2_S/CaP Nanoparticles

Ag_2_S/CaP (300 µg mL^−1^) was irradiated by an 808 nm laser with a power density of 1 W cm^−2^ for 5 min. Furthermore, the real‐time temperature was recorded using an infrared thermal imaging camera (FOTRIC 220s; FOTRIC Thermal Intelligence, China). To facilitate comparative analysis, the photothermal properties of H_2_O and Ag_2_S nanodots were recorded under the same experimental conditions. The photothermal performance of H_2_O, Ag_2_S, and Ag_2_S/CaP was examined by exposing them to an 808 nm NIR laser with varying power densities (0.5, 1.0, 1.5, 2.0 W cm^−2^), and the thermal images were captured.

### Cell Culture

B16F10 cells were sourced from the American Type Culture Collection (ATCC). Cells were cultured in DMEM enriched with 10% FBS, and 1% penicillin (50 U mL^−1^), and streptomycin (50 U mL^−1^) in a 5% CO_2_ atmosphere at 37 °C.

### Cellular Uptake and Lysosomal Escape

The B16F10 cells were seeded in confocal dishes and incubated with NR loaded‐Ag_2_S/Ca^32^P for specific time points (1, 2, 4, and 6 h). Afterward, the cells were stained by Hoechst 33342. Ultimately, images of the treated cells were captured with a confocal laser scanning microscopy (CLSM, ZEISS LSM900, Germany).

### Cell Viability Assay

The B16F10 cells were seeded in 96‐well plates overnight (2000 cells per well), and treated with various formulations for 48 h. After discarding the supernatant, MTT solution (5 mg mL^−1^) at a volume of 100 µL was added to each well for incubation for 4 h at 37 °C. Afterward, the absorbance at 570 nm was measured with a microplate reader (EPOCH‐SN, Biotek, US).

### Intracellular ROS Detection Assay

The B16F10 cells were seeded in 24‐well plates overnight (5 × 10^4^ cells per well), and treated with various formulations for 24 h. After incubation, cells were treated with the DCFH‐DA probe and incubated for 30 min under dark conditions. Subsequently, the cells were washed with PBS, and the generation of ROS was observed under an inverted fluorescence microscope (N2Ti2‐A, Leica, Germany).

### Immunofluorescence Staining

The B16F10 cells were seeded in 12‐well plates overnight (1.5 × 10^5^ cells per well), and treated with various formulations for 24 h. Then, cells were fixed with a 4% paraformaldehyde solution. Then, the cells were exposed to 0.2% Triton X‐100 for 20 min, followed by a 30‐min incubation in 10% goat serum to block non‐specific binding. Next, diluted primary antibody (γ‐H_2_AX) was added to the cells and cultured overnight at 4 °C. On the following day, the secondary antibody was added and incubated at 37 °C for 1 h. Finally, the cells were stained with DAPI and observed using an inverted fluorescence microscope.

### Live/Dead Cell Staining Assay

The B16F10 cells were seeded in 6‐well plates overnight (1.5 × 10^5^ cells per well), and treated with various formulations for 24 h. Subsequently, the cells were treated with Calcein‐AM/PI working solution for 30 min at 37 °C. Finally, the cells were harvested, centrifuged, and resuspended in PBS, then dropped onto glass slides and observed under an inverted fluorescence microscope.

### Preparation and Characterization of GM‐Ag_2_S/Ca^32^P Microneedle Patch

The obtained Ag_2_S/Ca^32^P suspension was mixed with a GelMA solution and LAP solution to form a 20% GelMA precursor solution containing 0.25% LAP. Then, the microneedle precursor solution was poured into a PDMS microtip mold. After defoaming under vacuum and centrifugating for three cycles, the tips were solidified through photo‐crosslinking under 405 nm UV light. Next, a 20% HA solution was applied to the bottom of the mold, and the defoam with the centrifugation process was repeated. Finally, the GM‐Ag_2_S/Ca^32^P microneedle patch was de‐molded from the PDMS mold and sealed under a dry environment for further application. The fluorescent‐labeled microneedle patch was fabricated by incorporating NR, RhB, or ICG‐labeled Ag_2_S/Ca^32^P into the precursor solution. In addition, the optical images of the microneedle patch were captured by a stereomicroscope (MZ62, Mshot, China). The needle surface morphology was observed using scanning electron microscopy (SEM, Phenom ProX G6, Netherlands), and the elemental distribution of the microneedle was determined by EDS (Phenom ProX G6, Netherlands). The mechanical strength of GM and GM‐Ag_2_S/CaP was measured using an electronic universal testing machine (CMT6103, China). The fluorescence images were obtained by the CLSM.

### In Vitro Anti‐Bacterial Performance

The in vitro antibacterial efficacy of the GM‐Ag_2_S/Ca^32^P microneedle patch was evaluated utilizing the spread plate method. First, the *S. aureus* suspension (100 µL, 1 × 10^8^ CFU mL^−1^) was incubated with microneedle patches of different formulations (Control, GM, GM‐Ca^32^P, GM‐Ag_2_S, GM‐Ag_2_S + Laser, GM‐Ag_2_S/Ca^32^P, GM‐Ag_2_S/Ca^32^P + Laser) at 37 °C for 30 min. Then, the Laser groups received irradiation from a NIR laser (808 nm, 1 W cm^−2^) for 5 min and continued to be cultured at 37 °C for 4 h. Ultimately, the suspension diluted with PBS was dispersed onto LB agar plates and incubated for 12 h. Finally, the bacterial colonies were counted.

### In Vivo Penetration Capability

Male C57BL/6 mice (6 weeks old, 20 ± 2 g) were obtained from the Department of Laboratory Animals of Central South University (Changsha, China). All procedures involving animals were authorized by the Animal Ethics and Welfare Committee of Central South University (2 024 090 918) and were conducted in accordance with the National Act on the Use of Experimental Animals (People's Republic of China).

After anesthetizing the mice, the skin on their backs was shaved and disinfected. Subsequently, the GM‐Ag_2_S/CaP microneedle patch was inserted into the back skin of the mice and removed at 10 min, 2, 4, and 12 h, respectively. The degradation of the microneedles was recorded using an optical microscope (Scope.A1, ZEISS, Germany). To further assess the skin recovery time, the microneedle patch was inserted into the dorsal skin of the mice. After 2 min, the microneedle patch was taken out, and the punctures on the skin were observed at 10 and 20 min with a digital camera (EOS700D, Canon, Japan). Simultaneously, the skin was collected and fixed with 4% paraformaldehyde for hematoxylin‐eosin (H&E) staining. To further monitor the insertion depth under the skin, RhB‐labeled microneedle patches were inserted into the skin on the dorsal of mice and removed after 2 min. Then, the skin was collected for a frozen section and observed with an inverted fluorescence microscope.

### In Vivo Accumulation and Biodistribution Behavior

For the accumulation and biodistribution behavior of GM‐Ag_2_S/Ca^32^P in vivo, 1 × 10^6^ B16F10 cells were subcutaneously injected into male C57BL/6 mice to establish the melanoma‐bearing mouse model. Once the volume of the tumor reached 50 mm^3^, an ICG‐labeled microneedle patch was applied to the tumor and removed after 2 h. The accumulation of ICG‐loaded Ag_2_S/CaP was observed through in vivo fluorescence imaging (S12‐FMT400010, PerkinElmer, US). For biodistribution, GM‐Ag_2_S/Ca^32^P (^32^P = 1 mCi kg^−1^) was inserted into tumor‐bearing mice. Subsequently, the mice were euthanized at predetermined time points (2, 24, 48, 72, and 96 h), and the radioactivity in the heart, liver, spleen, lung, kidneys, and tumor was quantified utilizing a gamma counter (WIZARD, PerkinElmer, US).

### In Vivo Antitumor Efficiency

For the antitumor effect study, the tumor‐bearing mice were randomly divided into seven groups and treated: PBS, GM, GM‐Ca^32^P, GM‐Ag_2_S, GM‐Ag_2_S + Laser, GM‐Ag_2_S/Ca^32^P, GM‐Ag_2_S/Ca^32^P + Laser. The group with NIR irradiation (808 nm, 1 W cm^−2^) was applied 5 min after microneedle patches were inserted. All radioactive treatment groups received a radiation dose of 2.5 mCi kg^−1^. The body weight and tumor volume were monitored every three days. For the purpose of survival study, the endpoint for monitoring was defined as either death or when the tumor volume reached 2000 mm^3^. On day 15 after treatment, the mice were sacrificed and the tumors were harvested for H&E and immunofluorescence staining (γ‐H_2_AX and TUNEL).

### In Vivo Tumor Recurrence Evaluation

To evaluate the efficacy of the microneedle patch in inhibiting postoperative tumor recurrence, the suspension of B16F10 cells (1 × 10^6^ cells mL^−1^) was inoculated on the right back of male C57BL/6 mice to establish a melanoma model. When the tumor volume reached 100 mm^3^, a circular full‐thickness skin defect (10 mm) was created at the tumor site, and ≈95% of the tumor tissue was resected. The mice were then randomly divided into seven groups and treated: PBS, GM, GM‐Ca^32^P, GM‐Ag_2_S, GM‐Ag_2_S + Laser, GM‐Ag_2_S/Ca^32^P, GM‐Ag_2_S/Ca^32^P + Laser. The group with NIR irradiation (808 nm, 1 W cm^−2^) was applied 5 min after the microneedle patches were inserted. All radioactive treatment groups received a radiation dose of 2.5 mCi kg^−1^. The body weight and tumor volume of mice were recorded every three days. Tumor volume was calculated as (tumor width)^2^ × (tumor length)/2. For the recurrence study, a tumor volume of 500 mm^3^ was considered tumor recurrence. After 30 days, the animals were euthanized, and tissues (lungs, liver, spleen, kidneys, heart, tumor) were harvested for H&E staining. Besides, serum was isolated to detect the biochemistry parameters.

### In Vivo *S. aureus*‐Infected Model Establishment and Histopathological Staining

A 10 mm diameter wound was created on the backs of C57BL/6 mice using a sterilized scalpel. Next, 20 µL *S. aureus* suspension (1 × 10^8^ CFU mL^−1^) was dropped to the wound to establish a wound infection model. After bacteria inoculation, microneedle patches were applied to the wound, with groups divided as follows: PBS, GM, GM‐Ca^32^P, GM‐Ag_2_S, GM‐Ag_2_S + Laser, GM‐Ag_2_S/Ca^32^P, GM‐Ag_2_S/Ca^32^P + Laser. All radioactive treatment groups received a radiation dose of 2.5 mCi kg^−1^. Body weight and wound size were documented on days 0, 1, 3, 5, 7, 9 and 12. Additionally, exudate from the wound was collected on days 0, 1, 3, 5, 7, 9, and 12 after treatment, diluted and spread onto agar plates to monitor bacterial colonies. Besides, the wound tissue of specific groups (PBS, GM, GM‐Ag_2_S/Ca^32^P, and GM‐Ag_2_S/Ca^32^P + Laser) was harvested for histopathological staining, including H&E, Masson Trichrome and immunofluorescence staining (TNF‐α, IL‐10, Sirius red and VEGF).

### Statistical Analysis

All statistical analyses were presented using GraphPad Prism 8 software. Quantified data were expressed as mean ± standard deviation (SD). The student's *t*‐test and one‐way ANOVA were utilized to determine whether data groups differed significantly from each other. Statistical significance was analyzed as follows: ^*^ *p* < 0.05, ^**^ *p* < 0.01, ^***^ *p* < 0.001.

## Conflict of Interest

Weibo Cai declares conflict of interest with the following corporations: Portrai, Inc., rTR Technovation Corporation, Four Health Global Pharmaceuticals Inc., and POP Biotechnologies, Inc. All other authors declare no conflict of interest.

## Supporting information



Supporting Information

## Data Availability

The data that support the findings of this study are available from the corresponding author upon reasonable request.
