# Supplementary material for: Biomineralized Nanocomposite‐Integrated Microneedle Patch for Combined Brachytherapy and Photothermal Therapy in Postoperative Melanoma Recurrence and Infectious Wound Healing
Source: Adv Sci (Weinh). 2025 Feb 4;12(12):2414468. doi: 10.1002/advs.202414468 (PMC11948049; doi:10.1002/advs.202414468)
Supplement: Supplementary file 1 — Supporting Information [file ADVS-12-2414468-s001.docx]

**Biomineralized Nanocomposite-integrated Microneedle Patch for Combined Brachytherapy and Photothermal Therapy in** **Postoperative Melanoma** **Recurrence and** **Infectious** **Wound Healing**

Peng Liu^1, 2, 3+^, Lu Hao^1+^, Jessica C. Hsu^3^, Ming Zhou^1^, Zhisheng Luo^1^, Ying Peng^4^, Weibo Cai^3^*, Shuo Hu^1, 2^*

^1^. Department of Nuclear Medicine, Xiangya Hospital, Central South University, No. 87 Xiangya Road, Changsha, Hunan 410008, China

^2^. Key Laboratory of Biological Nanotechnology, NHC. No. 87 Xiangya Road, Changsha Hunan 410008, China

^3^. Departments of Radiology and Medical Physics, University of Wisconsin-Madison, Madison, WI, 53705, USA

^4^. Xiangya School of Pharmaceutical Sciences, Central South University, Changsha, Hunan, 410013, China

[^+^] These authors contributed equally to this work.

*Email: wcai@uwhealth.org

hushuoxy@csu.edu.cn

**
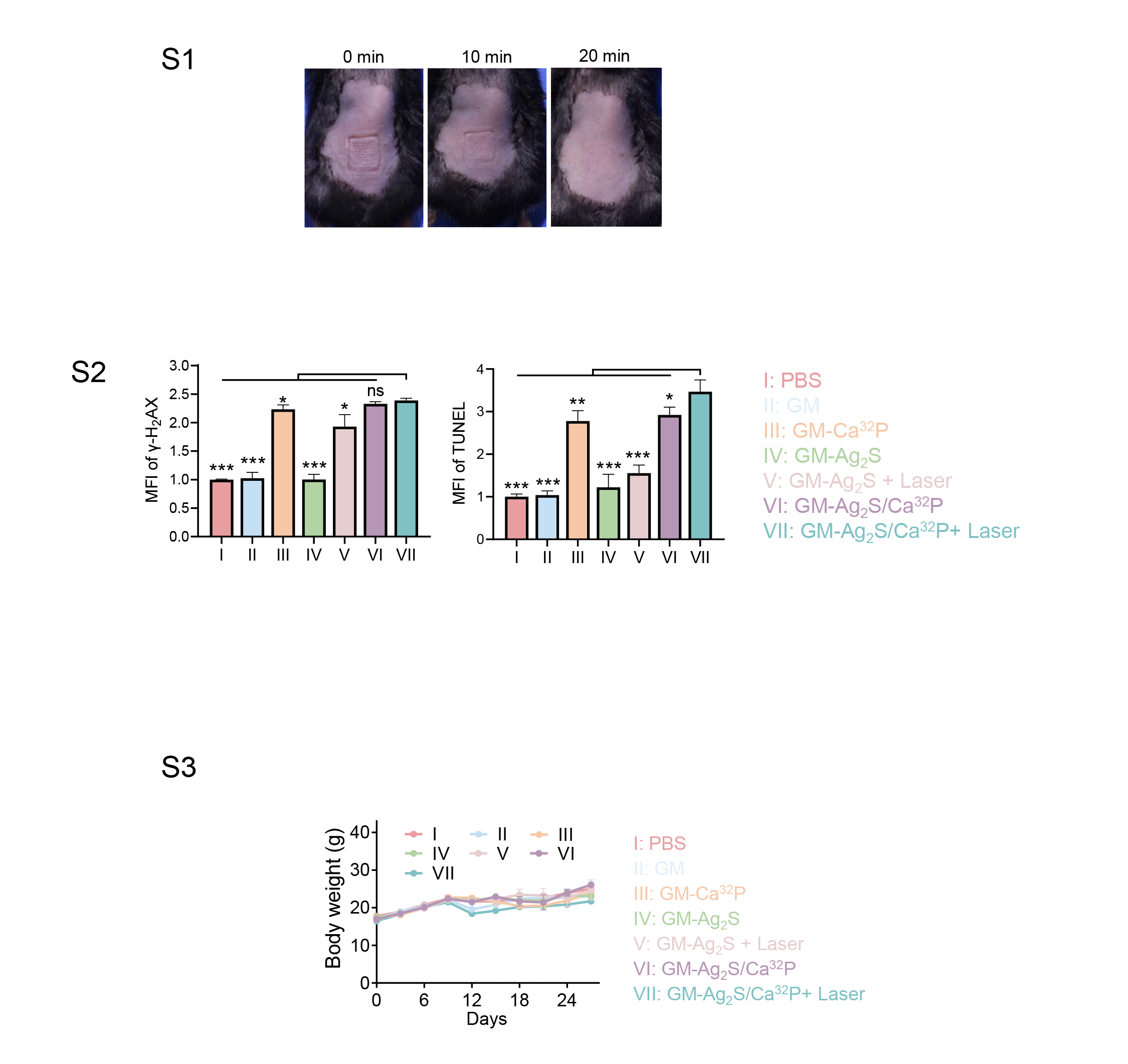
**

**Figure S1.** Photographs taken at various times after removing the microneedle patch.

**
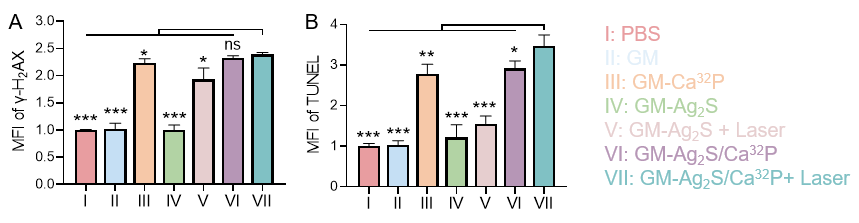
**

**Figure S2.** Quantitative analysis of (A) γ-H2AX and (B) TUNEL levels from Figure 4I.

**
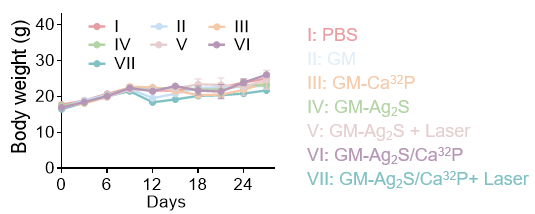
**

**Figure S3.** Average body weights of mice during various treatments in a postoperative melanoma model.

**
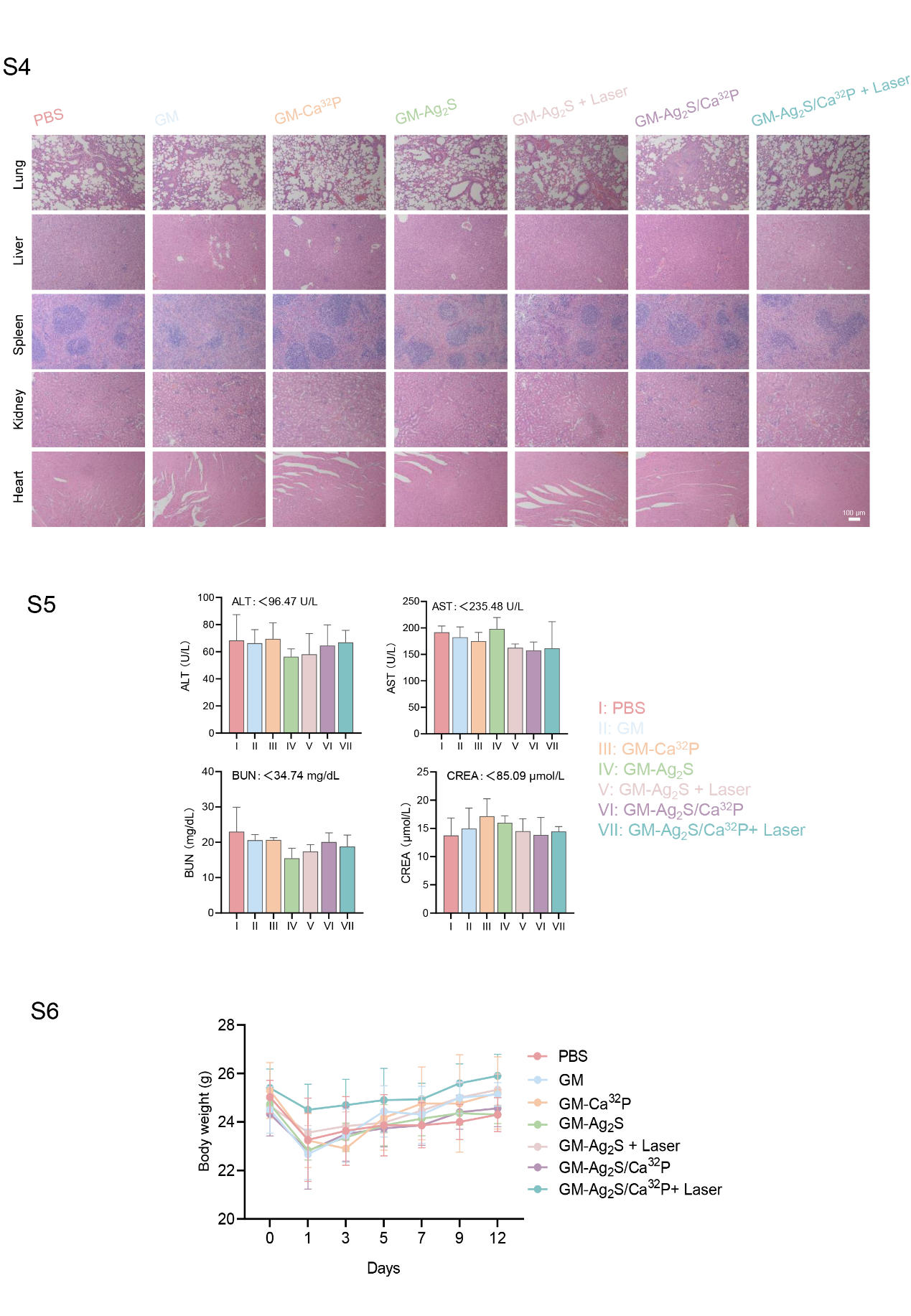
**

**Figure S4.** H&E staining of heart, kidney, spleen, liver and lung after different treatments.

**
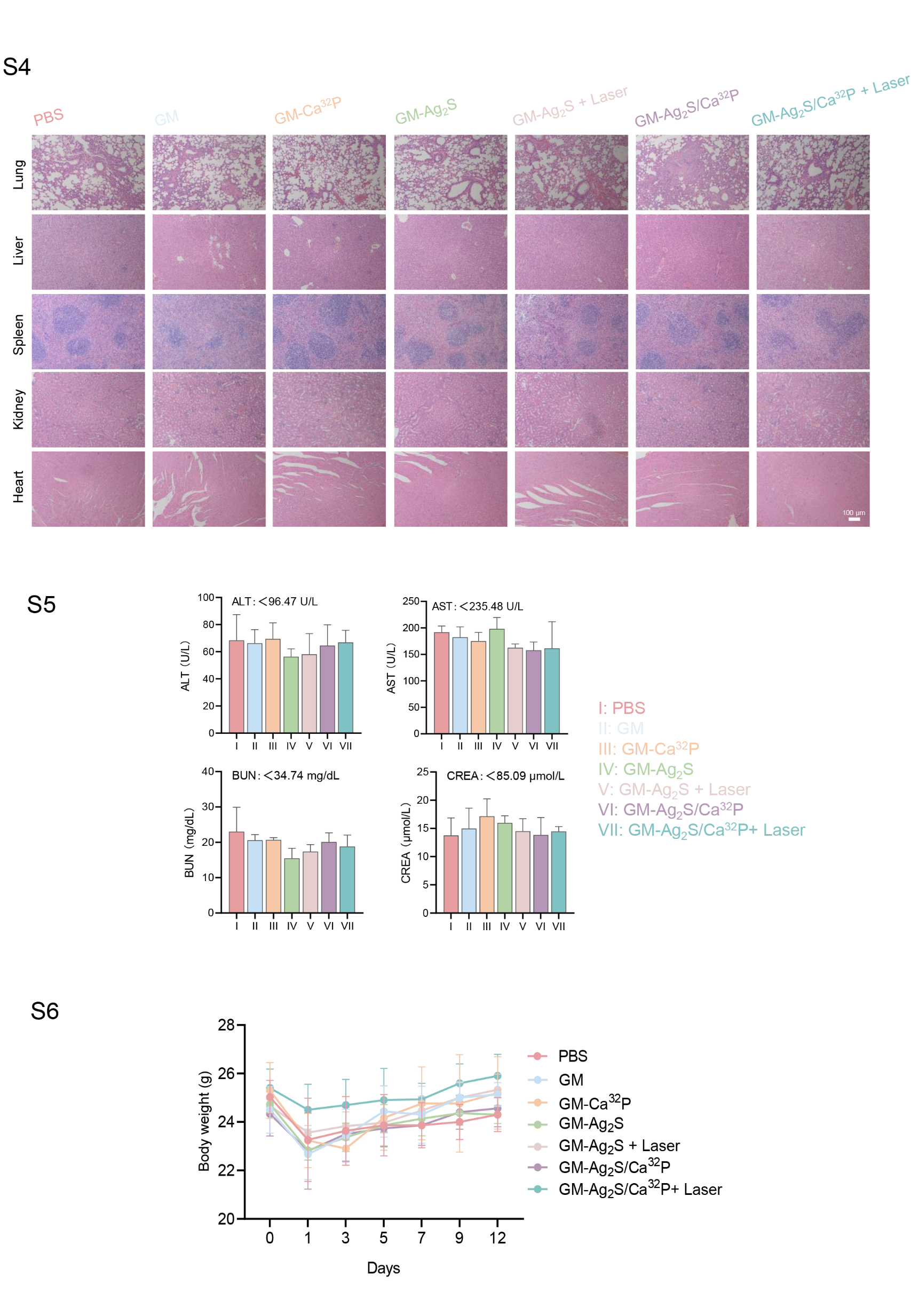
**

**Figure S5.** The serum levels of ALT, AST, BUN, and CRE of mice after various treatments.

**
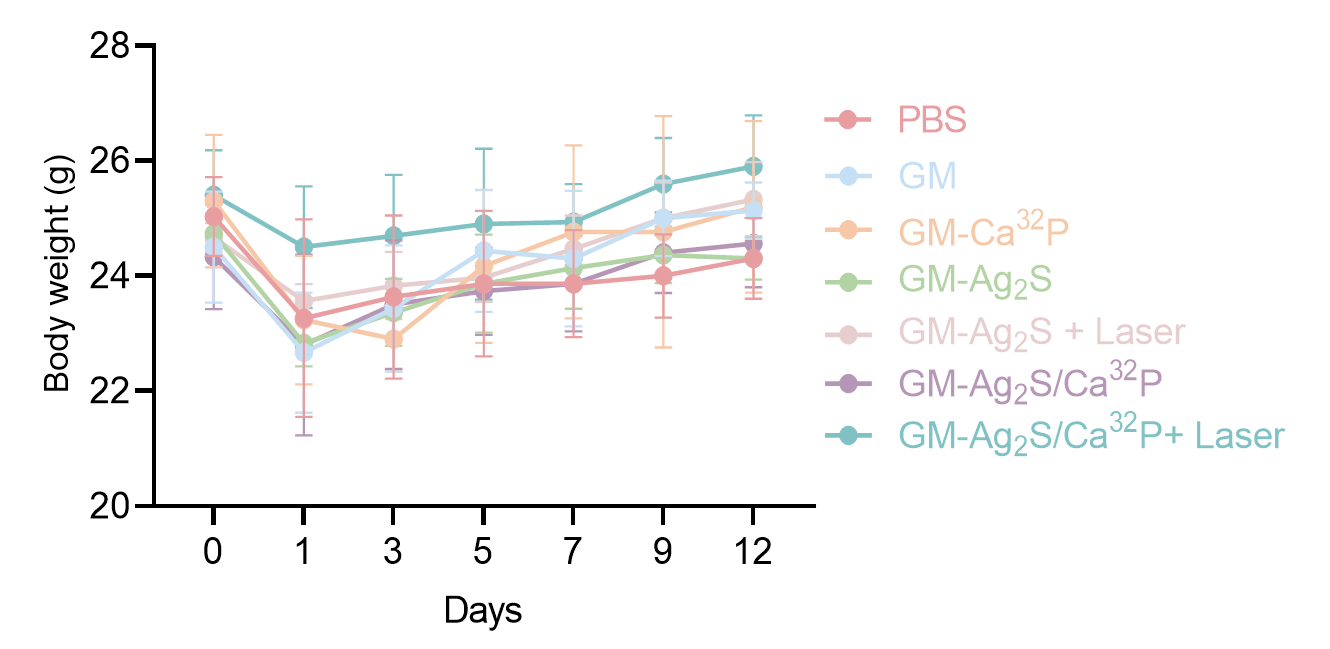
**

**Figure S6.** Average body weights of mice during various treatments in a full-thickness *S. aureus*-infected wound model.
